# Manipulating *Agaricus bisporus* developmental patterns by passaging microbial communities in complex substrates

**DOI:** 10.1128/spectrum.01978-23

**Published:** 2023-10-13

**Authors:** Fabricio Rocha Vieira, Isako Di Tomassi, Eoin O'Connor, Carolee T. Bull, John A. Pecchia, Kevin L. Hockett

**Affiliations:** 1 Department of Plant Pathology and Environmental Microbiology, The Pennsylvania State University, University Park, Pennsylvania, USA; 2 Microbiome Center, The Pennsylvanian State University, University Park, Pennsylvania, USA; 3 The Huck Institutes of the Life Sciences, The Pennsylvania State University, University Park, Pennsylvania, USA; Zhejiang University - Zhijiang Campus, Hangzhou, China

**Keywords:** *Agaricus bisporus*, compost, casing, peat moss, devome, microbiome manipulation

## Abstract

There is growing interest in microbiome-based management approaches for plant and animal health and sustainable agricultural practices. To our knowledge, there are no descriptions of *Agaricus bisporus* mushroom developmental patterns using such an approach. Here, we utilized substrate passaging to select for devomes (developmental microbiomes) in either compost or casing material that resulted in earlier developing (best performing experimental units with a higher number of primordia at day 10 of cultivation) mushrooms compared to unpassaged controls. Passaged casing showed earlier pinning (fruiting body formation), while passaged compost delayed fruiting body formation. Despite changes in pin emergence for passaged substrates and higher mushroom yield in the passaged casing’s first harvest, the total mushroom yield in 24 days of crop cycle did not change significantly. The bacterial communities were distinct between compost and casing materials, but in both microenvironments, the alpha diversity tended to increase and stabilize over time in standard and passaged substrates. Community composition within compost and casing microenvironments tended to be more homogenous after colonization of the fungus. Passaging of casing increased the bacterial alpha diversity and shifted community composition. Such changes were not observed in passaged compost. Some bacterial taxa were enriched or depleted by passaging, e.g., *Pseudomonas* spp. (hypothesized as a stimulus to *A. bisporus* fructification) were depleted in passaged compost and casing. Overall, this work presents an experimental approach to manipulate compost and casing microbial communities with the potential to be combined with currently employed cultivation strategies toward a more sustainable mushroom cultivation system.

**IMPORTANCE:**

*Agaricus bisporus* is an economically important edible mushroom and manipulating its developmental patterns is crucial for maximizing yield and quality. One of the potential strategies for achieving such a goal is passaging microbial communities in compost or casing. The current study demonstrated that passaging substrates develop enriched microbial communities, and after a few passages, certain levels of changes in mushroom developmental patterns (the timing of fruiting bodies formation) were observed as well as shifts in the bacterial communities. Overall, a better understanding of the complex interactions between microorganisms present in the cultivation system may help farmers and researchers to develop more efficient and sustainable cultivation practices that can both benefit the environment and human health.

## INTRODUCTION

The cultivation of mushrooms is a controlled agricultural process carried out in indoor settings year-round and is efficient in terms of yield per area cultivated. Several mushroom species are cultivated on a commercial basis but *Agaricus bisporus* (button mushroom) is preferred in Western countries, including Europe and North America (1 – 3). In the United States, the button mushroom economy supports dozens of farmers yielding 308,000 t during the 2021–2022 season, valued at $ ~930 million (4).

In nature, *A. bisporus* is a poor competitor in dead, non-degraded, non-woody material, and is a secondary decomposer in an organic-rich ecological niche. However, it is specifically adapted to grow on partially decomposed humic-rich plant material, commonly found in grassland ecosystems (5, 6). Different from other mushroom species (e.g., *Flammulina velutipes* and *Pleurotus ostreatus*) that can be cultivated in axenic conditions, the cultivation of *A. bisporus* relies upon selective substrates (compost and casing), and thus ecological relationships with a wide range of microorganisms present in the cultivation environment (7 – 9).

Microbiologically, the cultivation process mimics a selective organic-rich ecosystem that can be divided into two heterogeneous microbial microenvironments: the compost and casing layers (7, 9). The compost layer is a constructed medium based on a mixture of plant and animal materials transformed through an aerobic, two-phase, solid-state fermentation process (composting phases I and II) before becoming a substrate that selects for *A. bisporus* growth (7, 10, 11). For the casing layer, peat moss is often chosen as the main material. Like compost, it originated from a solid-state fermentation process, but occurs naturally in wetlands in an acidic and anaerobic environment (“peat moss bogs”). In contrast to the compost that provides nutrition for the fungus, the casing layer provides a hydrated, low-nutrient environment suitable for fruiting body development (2, 7).

Each of the compost and casing layers carry distinct microbial communities and this assembled and interconnected microbial network is one of the critical factors in modulating mushroom developmental patterns under cultivation practices (12, 13) and likely functions as a devome (developmental microbiome; 14). Devomes have been proposed in many systems, but because of its unique features, the mushroom cultivation system lends itself to studying a devome *in situ*. For example, microbial manipulation, populational enrichment of native microorganisms can significatively impact *A. bisporus* growth behavior. For example, faster vegetative growth was observed when the fungus *Mycothermus thermophilus* was inoculated as a biostimulant in compost (15, 16); changes in initiation/formation patterns of primordia (“baby mushrooms”) were observed following inoculation of *Pseudomonas putida* in the casing (17 – 19).

In particular, we lack a full understanding of the taxa present in either casing or compost environments that participate in the devome either by promoting or inhibiting mushroom formation. To identify such microbial taxa, and thus develop a more robust microbiological management model, we utilized a substrate passaging approach (addition of colonized compost and casing to fresh substrate in a ratio of 1:10) to select for either compost or casing material that resulted in earlier developing (best performing experimental [pots] units with higher number of primordia at day 10 of cultivation) mushrooms compared to non-passaged controls. Once selected, we used 16S rRNA gene amplicon sequencing to identify those bacterial populations that were specifically enriched or depleted in the passaged substrates compared to the control compost and casing.

## RESULTS

**Fig 1:**
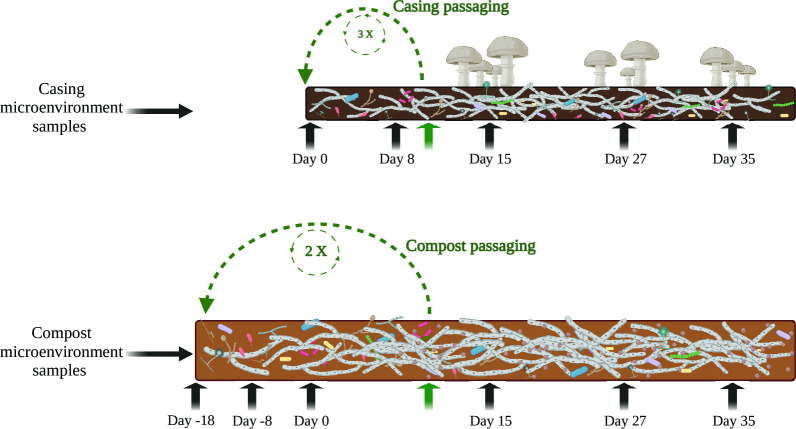
Overview of passaging design to select for devomes (developmental microbimes) in compost and casing materials used in the cultivation of *Agaricus bisporus*. Compost samples (lower portion of the diagram): day −18 – sample at the end of composting phase II in the original crop; day −8 – sample from the middle of spawn run (day 8 after spawning) for either standard or passaged compost treatments; day 0 – sample at the end of spawn run (18 days after spawning) for standard and passaged compost; day 15 – sample from the first flush for standard and passaged compost; day 27 – sample from the second flush for standard and passaged compost; day 35 – sample from the third flush for standard and passaged compost. Casing samples (upper portion of the diagram): day 0 – mixed peat moss, limestone, and case inoculum; day 8 – sample from the middle of casehold for standard and passaged casing; day 15 – sample from the first flush for standard and passaged casing; day 27 – sample from the second flush for standard and passaged casing; day 35 – sample from the third flush for standard and passaged casing. The green arrows represent the points at which material was collected for subsequent passaging in both the compost and casing.

**Fig 2:**
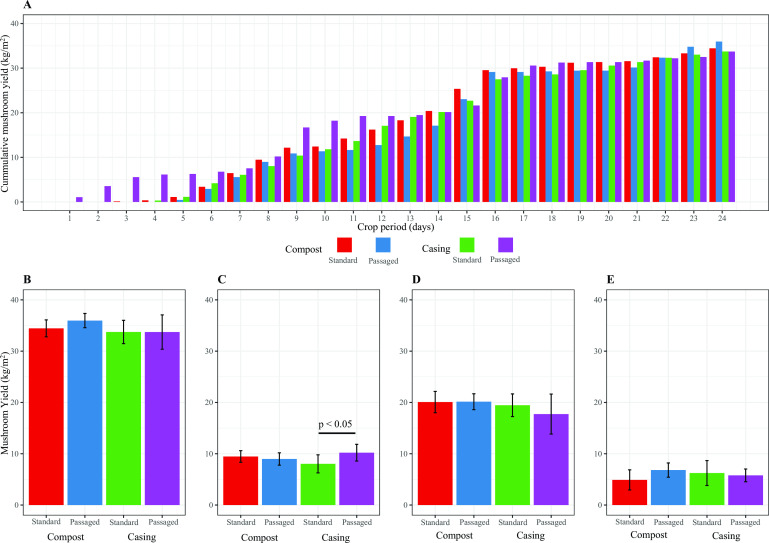
A – Cumulative mushroom yield during 24 days of mushroom harvest. B– Total mushroom yield (three flushes). C– Mushroom yield for the first harvest (first flush). D– Mushroom yield of second harvest (second flush) and E– mushroom yield of third harvest (third flush). Passaged casing has a significant impact on mushroom yield only at the first flush (*P* < 0.05, Tukey test).

The passages were carried out separately for both microenvironments, colonized compost with *A. bisporus* mixed with an uncolonized compost and colonized casing mixed with an uncolonized casing (Fig. 1). Colonized compost and/or casing (passaging inoculum) were collected during the primordia initiation period (mushroom pinning stage, day 10 of case hold) and was repeated twice for compost and three times for casing to prepare the enriched materials before using in a crop that was allowed to mature to collect yield data. The impact of passaging on mushroom development was measured based on pinning time (the time it takes to form fruiting bodies from casing day 0) and mushroom yield (kilograms of fresh mushrooms per square meter of a compost bed). During the 24-day period of mushroom harvesting (3 flushes of 8 days each), passaged compost was shown to delay (1.5 days) the pinning stage at the first flush, but it did not impact mushroom yield at the first flush (*P* > 0.05, Fig. 2A and B). On the other hand, a slight increase in mushroom yield (total yield, *P* > 0.05) was observed at the end of the crop period for the passaged compost (Fig. 2A and B). Oppositely, passaged casing promoted early pinning initiation (3 days) when compared with standard casing (*P* < 0.05, Fig. 2A and C). Despite observed changes in mushroom pinning patterns in compost and casing microenvironments, late crop harvesting (first and second flushes) did not show significant yield differences (*P* > 0.05) (Fig. 2D and E). The total mushroom yield (three flushes) for both microenvironments was similar (*P* > 0.05) when compared with the control treatments (Fig. 2B).

The bacterial communities of both microenvironments were evaluated based on community snapshots generated by direct genomic DNA (gDNA) recovered from compost and casing samples from the major crop stages for both treatments—standard and passaged (Fig. 1). After sequence processing, more than half of the raw reads (59.5%) remained for taxonomic assignment (Table S1). In the mock community, eight sequence variants (SVs) were recovered with a matching score of 100% with reference sequences (data not shown). SVs that do not belong to the Bacteria kingdom (archaea, one SV compressing two sequences) were excluded from the data set, as well as SVs classified as Bacteria only (356 SVs compressing 2,739 sequences), candidate phyla (WPS-2, 17 SVs compressing 4,348 sequences) and mitochondria (48 SVs compressing 489,677 sequences) (Table S2). The remaining SVs (compressing a total of 3,488,632 sequences) were then divided by microenvironments and rarefied for some downstream analyses, *n* = 25,957 for compost and *n* = 40,024 for casing (Tables S3 and S4). Sequencing depth showed a higher coverage trend for uncolonized samples (compost and/or casing) than colonized samples (Fig. S1). For compost samples, the percentage of sequences classified below phylum decreased as follows: class 99.8%, family 88.2%, genus 61.2%, and species 15.5% (Table S3); and for casing samples: class 99.7%, family 96.4%, genus 86.8%, and species 7.6% (Table S4).

**Fig 3:**
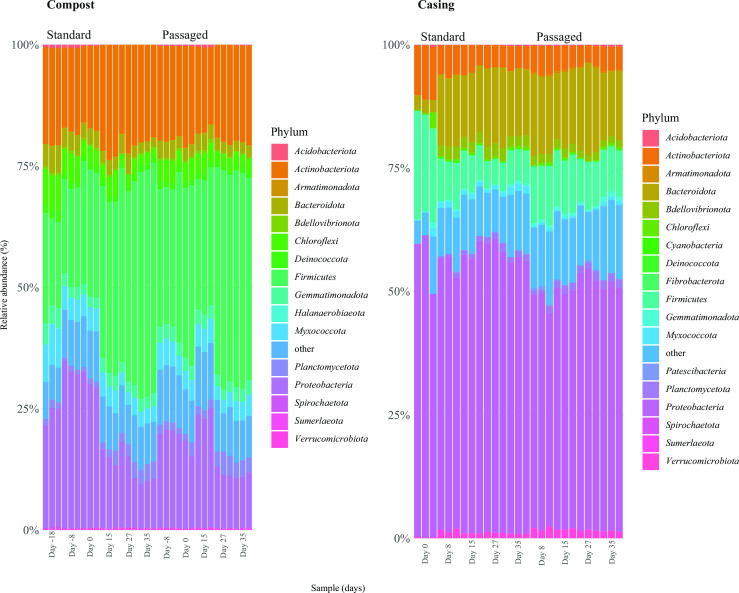
Relative abundance of sequences at the phylum level grouped by microenvironment and distributed by crop stages. Compost samples: day −18 – sample at the end of composting phase II in the original crop; day −8 – sample from the middle of spawn run (day 8 after spawning) for either standard or passaged compost treatments; day 0 – sample at the end of spawn run (18 days after spawning) for standard and passaged compost; day 15 – sample from the first flush for standard and passaged compost; day 27 – sample from the second flush for standard and passaged compost; day 35 – sample from the third flush for standard and passaged compost. Casing samples: day 0 – mixed peat moss, limestone, and case inoculum; day 8 – sample from the middle of casehold for standard and passaged casing; day 15 – sample from the first flush for standard and passaged casing; day 27 – sample from the second flush for standard and passaged casing; day 35 – sample from the third flush for standard and passaged casing.

A total of 24 phyla were detected in both microenvironments (Fig. S2; Table S3 and S4). Among these phyla, sequences that belong to Firmicutes were the most abundant in compost, followed by Proteobacteria, Actinobacteriota, and Bacteriodota (compressing 82.7% of the total sequences, Fig. 3; Table S3). Sequences of Firmicutes appear to increase when *A. bisporus* is at the reproductive stage (fruiting body formation, samples during harvesting period) and this trend was not as pronounced in passaged compost samples (Fig. 3). In casing, the most abundant sequences were of Proteobacteria followed by Bacteriodota, Firmicutes, and Actinobacteriota (compressing 90.7% of the total sequences, Fig. 3; Table S4). Conversely for compost, less fluctuation in relative abundance of phyla was observed between standard and passaged treatments. It is worth noting that some phyla appear to have specificity for sequence distribution patterns regarding these specific microenvironments, e.g., a higher number of sequences of Firmicutes in compost while casing showed a higher number of Proteobacteria sequences (Fig. 3).

**Fig 4:**
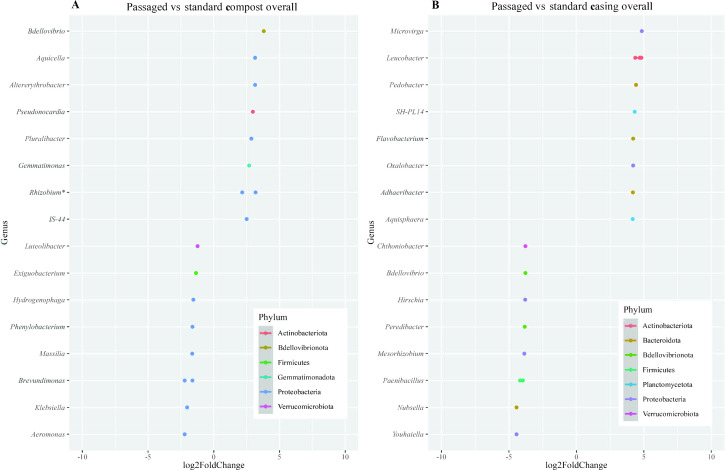
Differential abundance analyses for both microenvironments. A – Passaged vs standard compost and B – passaged vs standard casing. The figure displays the most differential genera (a total of 18 in each microenvironment). Information about other genera can be found in Table S5 and S6.

Approximately 1,325 SVs (438 unique genera, compressing 524,825 sequences) were identified at the genus level in compost (Table S3), and when treatments (passaged vs standard) were compared, some genera (18 SVs in total) showed significant differential abundance (*P* < 0.01) between the treatments (Fig. 4A; Table S5). In casing, 1,323 SVs (460 unique genera, compressing 887,554 sequences) were identified at the genus level and the number of genera that shifted in abundance (*P* < 0.01) was higher than compost when samples were sorted by treatments, 453 genera between passaged and standard casing (Fig. 4B; Table S6).

**Fig 5:**
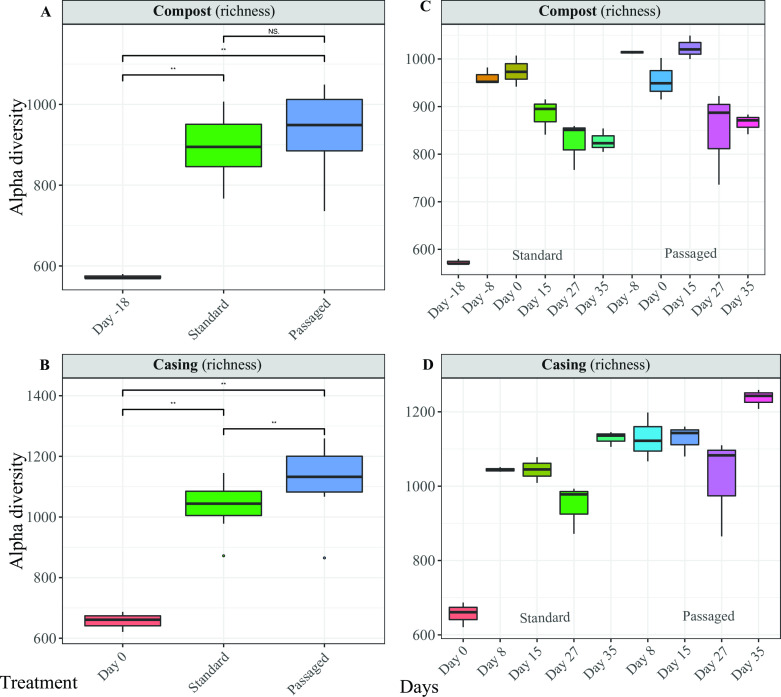
Alpha diversity index, samples grouped by microenvironments (A – compost and B – casing), treatments (A – compost and B – casing), and crop stages within each microenvironment (C – compost and D – casing). Compost samples: day −18 – sample at the end of composting phase II in the original crop; day −8 – sample from the middle of spawn run (day 8 after spawning) for either standard or passaged compost treatments; day 0 – sample at the end of spawn run (18 days after spawning) for standard and passaged compost; day 15 – sample from the first flush for standard and passaged compost; day 27 – sample from the second flush for standard and passaged compost; day 35 – sample from the third flush for standard and passaged compost. Casing samples: day 0 – mixed peat moss, limestone, and case inoculum; day 8 – sample from the middle of casehold for standard and passaged casing; day 15 – sample from the first flush for standard and passaged casing; day 27 – sample from the second flush for standard and passaged casing; day 35 – sample from the third flush for standard and passaged casing.

As one of the community diversity measurements, alpha diversity index (richness) was used to compare samples grouped by treatments (uncolonized samples were included as a treatment) and by crop stages within the same microenvironment. The alpha diversity index in colonized compost increased significantly compared with uncolonized compost (Fig. 5A). Passaged compost did not differ significantly from standard compost when grouped by treatments (Fig. 5A). For the same crop stage between treatments, no significant differences were observed in compost (Fig. 5C). It is worth noting that after casing passaged compost, the alpha diversity index tended to be higher at the first flush which during the second and third flush shifted down following the trend of standard compost. In the casing microenvironment, the alpha diversity index also increased significantly after colonization of *A. bisporus* (Fig. 5B). In contrast to compost, when samples were grouped by treatments, passaged casing had a higher alpha diversity index (*P* < 0.05) compared with standard casing. However, when samples were grouped by the same crop stage between treatments, no significant differences were observed for casing (Fig. 5D). However, passaged casing showed a slightly higher diversity index for all crop stages between different treatments (Fig. 5D).

**Fig 6:**
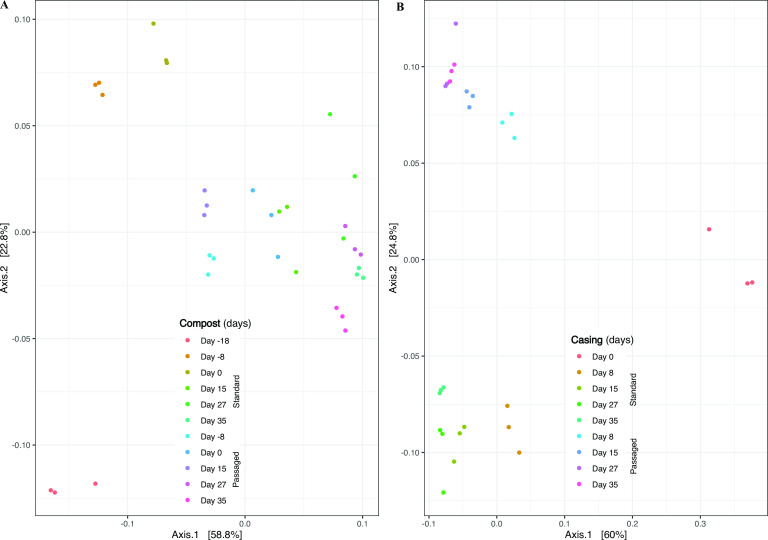
Beta diversity index, samples grouped by microenvironments and crop stages. A – Compost samples: day −18 – sample at the end of composting phase II in the original crop; day −8 – sample from the middle of spawn run (day 8 after spawning) for either standard or passaged compost treatments; day 0 – sample at the end of spawn run (18 days after spawning) for standard and passaged compost; day 15 – sample from the first flush for standard and passaged compost; day 27 – sample from the second flush for standard and passaged compost; day 35 – sample from the third flush for standard and passaged compost. B – casing samples: day 0 – mixed peat moss, limestone, and case inoculum; day 8 – sample from the middle of casehold for standard and passaged casing; day 15 – sample from the first flush for standard and passaged casing; day 27 – sample from the second flush for standard and passaged casing; day 35 – sample from the third flush for standard and passaged casing.

Beta diversity index was calculated using UniFrac (weighted) distance and ordinations were generated using principal coordinate analysis (PCoA) (Fig. 6A and B; Fig. S3 through 10). For statistical comparisons, samples were grouped by treatments (uncolonized samples included) and crop stages within the same microenvironment. In the compost microenvironment, when samples were grouped by treatments, uncolonized compost was significatively different (pairwise adonis, *P* < 0.05) from passaged and standard compost. Between passaged and standard compost, no differences (*P* > 0.05) were observed, as well as for crop stages within the same treatment or same crop stage between treatments. After the inoculation of *A. bisporus*, compost samples tend to be grouped together, except standard compost from early colonization stages (days −8 and −18 of spawn run) (Fig. 6A). In the casing microenvironment, when samples were grouped by treatments, pairwise adonis were significatively different (*P* < 0.05) between uncolonized casing and passaged and between passaged and standard. Like uncolonized compost, uncolonized casing was grouped separately from colonized casing. In contrast to compost, samples from different treatments were grouped separately from each other (Fig. 6A and B). Between crop stages within the same treatment or the same crop stage between treatments, no significant differences (*P* > 0.05) were observed.

The core community was calculated by treatments (standard and passaged) using a threshold of 1% relative abundance and 50% prevalence, i.e., SVs that are present in at least 50% of the samples with a relative abundance above 1%. In compost, the core community in standard compost was represented by 14 and 12 SVs in passaged compost. Between these SVs, 11 SVs were shared between both treatments while 3 were unique in standard compost and 1 and passaged compost (Table S6). In casing, 17 SVs were detected in standard casing and 19 in passaged casing with 8 shared between treatments and 9 unique in standard casing and 11 in passaged casing (Table S7).

## DISCUSSION

*A. bisporus* cultivation systems are excellent model devomes because the cultivation systems rely on non-sterile substrates (compost and casing), microorganisms native to the cultivation system cohabit and interact with the fungus, and such ecological relationships have been speculated as important driving factors for mycelial growth and fruiting body formation and development (12, 13, 20). The results outlined here provide some clues and may help growers and researchers to design strategies to manipulate devomes in the *A. bisporus* cultivation system, aiming to maximize the mushroom yield or manipulate the timing of yield.

In this study, passaging of substrate microbial communities (Fig. 1) impacted the fruiting bodies’ developmental patterns during the cultivation process. Passages of casing showed earlier fruiting body formation (Fig. 2A) with a significantly higher mushroom yield in the first flush (Fig. 2C). Oppositely, passages of compost delayed (later pinning) fruiting body formation (Fig. 2A) without a significant impact in the first flush (Fig. 2C). Despite some differences in the frequency of fruiting bodies formed during the crop cycle of 24 days (three flushes, Fig. 2C and D), the total mushroom yield was not impacted by passages of compost or casing (Fig. 2B). It is worth noting that fragments of *A. bisporus* mycelium were transferred with passages of compost and casing which resulted in faster substrate colonization (visual observations) but speeding up colonization does not necessarily reflect in an earlier fruiting body formation, e.g., passaged compost. The total mushroom yield is perhaps related to the availability of resources and not with time of colonization. Previous reports hypothesized that the pinhead’s nutrition may come from compost and have a relation with demand and not supply, i.e., the number of pinheads formed is smaller than the number of fruiting bodies that outgrow them and become pickable (13, 21).

The bacterial community has phyla distribution specificity between compost and casing, and phyla distribution changes within each microenvironment over time (Fig. 3). Sequences of Bacteriodota in casing increased rapidly after day 0 and became the second most abundant phylum, composing 1/4 of assigned sequences (Fig. 3; Table S4). Using a similar approach (i.e., amplicon sequencing), Vieira and Pecchia (9) reported similar results, i.e., phyla specificity between compost and casing and a rapid increase in sequences of Bacteriodota in casing after day 0; Carrasco et al. (22) reported Bacteriodota as the second most abundant phylum in the casing without significant shifts over time (after day 0). In compost, shifts in phyla distribution were less prominent than in casing. Passages of compost and casing slightly shifted in phyla distribution.

Examining bacterial genera distribution (Table S3 and S4), a similar number of unique genera in each microenvironment (compost 438 and casing 460) was observed and passaged casing had a higher number of genera shifted in abundance, 18 in compost and 453 in the casing (Fig. 4A and B; Table S5 and S6). Members of Proteobacteria were the most affected by passages in both microenvironments (Fig. 4A and B; Table S5 and S6). The phylum Proteobacteria host the genus *Pseudomonas*, one of the few well-known groups of bacteria present in the casing which act as a promoter of *A. bisporus* fruiting body formation (13, 19). At the same time, some species of *Pseudomonas* can act as a pathogen in *A. bisporus* mushroom caps (23, 24). The genus *Pseudomonas* was represented as a member of the core community in casing and passages of casing decrease their relative abundance compared with standard casing (Table S6). With less knowledge regarding their role in the cultivation system, *Pedobacter* and *Flavobacterium* were represented in the core community and were also depleted by the casing passages as well. Recent microbiome studies have demonstrated that these two genera are abundant in compost and casing materials used to cultivate *A. bisporus* but very little is known about their relationship with the fungus or with other members of the microbiome (8, 9). Most of the genera identified in the current study or in other recent studies using amplicon sequencing have not been examined yet. We currently lack knowledge about their ecological roles in the *A. bisporus* cultivation system.

In terms of community diversity, in compost (day −18) and casing (day 0) after inoculation of *A. bisporus,* a rapid increase was observed (Fig. 5A through D) and previous reports show similar trends, e.g., bacterial community alpha diversity tends to increase after the colonization of *A. bisporus* (9, 22, 25). With further examination, results from these studies show that bacterial community alpha diversity tends to increase over time, but the intensity and degree of these changes in community diversity may vary. Interestingly, passages of substrates showed to increase the community diversity in both microenvironments with a greater effect on the casing (Fig. 5A through D). The increase in diversity (richness) after day 0 for compost or casing raises the possibility that bacteria can be added to substrates (compost or casing) by the cultivation environment (e.g., *A. bisporus* inoculum, water in irrigation, tools, etc.) or after a certain threshold, these populations become detectable by the experimental techniques used here. In either hypothesis, the inclusion of control treatments (i.e., compost and casing without inoculation of *A. bisporus*) for future studies may help clarify which of these hypotheses should move forward.

Like community diversity, communities’ composition in compost and casing shifted in composition from uncolonized substrates (days −18 and 0 for compost and casing, respectively) to after the colonization of *A. bisporus* (Fig. 6A and B). These results are in accordance with other authors (20, 25), i.e., the bacterial communities tend to be more homogenous in later cultivation crop stages (harvesting period) for either compost or casing. Passages of compost have less impact on community composition which tends to be homogenous over time including passaged samples. In casing, the community composition also tends to be homogenous over time but in a different way than compost, passaged casing tends to shift in composition compared with standard casing.

The present study was designed to select, by successive passages, for either compost or casing material that resulted in earlier developing (using best performing experimental units, i.e., phenotype selection) mushrooms compared to unpassaged controls. Passages of microbial communities display potential as a tool to manage fruiting body formation frequency in the *A. bisporus* cultivation system. Despite compost and casing (peat moss) being organic-rich environments, these materials have different functions in the cultivation process as well as distinct bacterial communities. Passages of microbial communities appear to speed up the compost and casing colonization but did not necessarily produce more mushrooms. The results outlined here show that earlier fruiting body formation is achievable with passaged casing, and this may be related to the transfer of bacterial communities in which some members are known to play an important role during *A. bisporus* fruiting body formation.

## MATERIALS AND METHODS

### *Agaricus bisporus* cropping trials

The cropping trials were carried out using standard procedures at the Mushroom Research Center (https://plantpath.psu.edu/research/centers/mushroom-research-center) at Pennsylvania State University, PA, USA. A full crop process consists of three major steps: phase I and II composting; compost/casing (spawn run/casehold) colonization; and mushroom fructification and harvests. Details of cropping procedures such as compost formula, composting procedures, and environmental control can be found in Vieira and Pecchia (9). The ratio of 3% *A*. *bisporus* off-white strain 901 Lambert (Lambert 901 SI, PA, USA) spawn and 4% of commercial supplement (Promycel gold [54% protein] – Amycel, CA, USA) were used. Spawn and supplement ratios were based on dry weights of PII compost. For substrate colonization (spawn run), 3.6 kg (wet weight) of spawned and supplemented compost (standard or passaged) were transferred to rectangular PVC containers (23 cm height, 23 cm length, and 18 cm width) before being transferred to growing rooms. The spawn run was carried out with temperature of compost maintained at 24–25°C and air humidity of 95%, until the substrate was colonized (16 days). Sphagnum peat moss (Scott’s sphagnum peat moss, Ontario, CA) was mixed with crushed agricultural limestone to raise the pH to near 8.0, additional *A. bisporus* mycelium (casing inoculum) and water (to near saturation) were added prior to placing on top of the colonized compost. Approximately 1.4 kg of peat moss (casing) with moisture adjusted to ~75% was placed on top of colonized compost, forming a 5 cm layer. After casing application, the crop was watered by hand, as needed, and fruiting was induced following standard MRC growing protocols. Mushroom harvesting was initiated on day 15 after casing. A total of 24 days of harvesting was recorded (from the first mushroom picked to the end of the crop). Mushrooms were picked in three harvest cycles (flushes) with a duration of 8 days each. Mushrooms were harvested based on their maturity (2–4 cm in diameter) and were weighed for yield calculations. Mushroom yield represents kilograms of fresh mushroom per square meter of casing surface, for total yield (all flushes together) or by flush (1, 2, and 3). For statistical comparisons, normality test (Shapiro-Wilk) and Q-Q plots of the residuals were performed using stats R package version 4.2.1 (R Core Team 2022), which showed a normal distribution for the data set, and a parametric test (Tukey test) was used.

### Passaging approach

The passaging approach was based on transferring an assembled microbial community from colonized compost or casing when *A. bisporus* was at the development stage of hyphal knots, which then developed into “primordia” (Fig. 1). This timepoint occurred around day 10 after casing for each passaged crop and 30% of the best performing experimental units (pots) were chosen for the next rounds of passages. Samples (colonized compost or casing) were collected and placed in plastic bags and stored in a refrigerator at 4°C for up to a week until the next crop was ready for use. The second round of passaging was initiated by mixing a ratio of 10% (wet weight) of colonized compost/casing with fresh compost (phase II compost) or fresh casing (peat moss mixed with limestone and casing inoculum), respectively. The passaging process was repeated two cycles for compost and three cycles for casing. During these rounds of passaging, no yield was recorded, and the experimental units were destroyed providing material for the next round of passaging. After two cycles of passaging for compost and three cycles of passaging for casing, a full crop was carried out for mushroom development assessment (pinning time and mushroom yield) and bacterial community profiling.

### Sampling, DNA extraction, and sequencing

To access the bacterial community dynamics during the cultivation process, samples were taken from both microenvironments (compost and casing) during a full crop trial (passaged full crop), and each sample represented a “community snapshot” at that time point (Fig. 1). For some analyses, fresh compost (compost day −18) or fresh casing (casing day 0) was grouped as a treatment. Compost samples were collected at six time/points, at the end of phase II composting (day −18), in the middle of the spawn run (day −8), at the end of the spawn run (day 0), and in the middle of the first (day 15), second (day 27) and third (day 35) flushes (Fig. 1). Casing samples were collected at five time/points, casing day (day 0) in the middle of casehold (day 8), and in the middle of the first (day 15), second (day 27), and third (day 35) flushes (Fig. 1). Samples were collected from destructive experimental units (containers with compost and casing), i.e., three independent experimental units were destroyed for each time/point sampled and used as individual samples for gDNA extraction, library construction, sequencing, and analyses. From each experimental unit, compost samples were taken from the center of the compost layer (center of the compost block). The same was carried out for casing samples, i.e., casing samples were taken from the center of the casing layer for each experimental unit. All samples were collected and immediately stored at −80°C until gDNA extraction. After removing from storage (10 g wet), samples were freeze-dried and manually homogenized into a fine powder; 50 mg of the fine powder was used for gDNA extraction using a FastDNA Spin Kit for Soil (MP Biomedicals, OH, USA) following the manufacturer’s instructions. Positive control samples were included during the library construction, which consisted of gDNA of a commercial mock community (ZymoBiomcs Microbial Community DNA Standard, CA, USA) with eight species of bacteria. The concentration of gDNA was determined using Qubit dsDNA Assay Kit and visualized by agarose gel electrophoreses (agarose 1%, voltage 150 V for 45 minutes). Fragments of gDNA were end-polished (addition of A tailing) and amplified with primers (515F and 806R, 26) targeting the V4 region of the 16S rRNA gene. After, PCR products were purified using Agencourt AMPure XP beads (Beckman-Coulter Life Sciences, CA, USA) followed by a qualification using Agilent 2100 bioanalyzer prior being pooled and sequenced using a 250 PE Kit in a NovaSeq 6000 Illumina platform at the Novogene Corporation Ltd.

### Sequence processing and bioinformatics analysis

The resulting read files (FASTQ files) were processed using the DADA2 R package version 1.20.0 (27) in RStudio version 2022.7.1.554 (28) and R version 4.2.1 (29). Raw reads were checked for ambiguity (zero *N*s were allowed) and primer removal (forward and reverse) was performed by cutadapt command-line tool version 2.8 (30) as a plugin in DADA2. After, pre-processed reads were inspected for their quality score and reads with <*Q* 30 were removed from the data set. Qualified reads were then merged, with a read average length of 253 bp (length threshold chosen for the V4 region was 250–260 bp and sequences shorter or longer were removed). After chimera removal, the remaining sequences were aligned using a naïve Bayesian classifier method (31) against Silva 16S rRNA gene database version 138.1 (32). The DADA2 output files (taxonomic assignment and abundance of sequences) were manipulated using phyloseq R package version 1.40.0 (33). The sequencing and data processing “evaluation of accuracy” were carried out by simply checking the presence and absence of sequences in the positive samples (commercial mock community). The data set was pruned, and classified sequences only at the bacteria domain (i.e., no taxonomic classification at the phylum level and beyond) were removed prior to some downstream analyses. Most of the analyses were carried out using unrarefied and rarefied data for comparison and were discussed if necessary. To facilitate the display of results, candidate phyla were removed as well. Data sets rarefied or not and pruned or not can be accessed in the supplemental materials (Table S2 and S4). Taxa distribution, community profile, core community, and taxa prevalence calculations and visualization were carried out using R packages, MiscMetabar version 0.23 (http://github.com/adrientaudiere/Miscmetabar), microbiome version 1.18.0 (34), and phyloseq. Alpha diversity index was calculated using richness (observed reads) to compare samples grouped by treatments (standard and passaged) within each microenvironment and samples grouped by crop stages within the same microenvironment between treatments or within the same treatment. The calculations, plots, and statistical analyses (Wilcoxon test) were generated using MicrobiotaProcess R package version 1.8.1 (35). Beta diversity index was calculated using weighted UniFrac (36), grouping the samples in the same way as alpha diversity index. The ecological metric was visualized using PCoA plots with phyloseq and ggplot2 R package version 3.3.6 (37). Analysis of variance for beta diversity indices was performed using Adonis and pairwise PERMANOVAs with vegan R package version 2.6–2 (38). Differential abundance analysis was carried out using the DESeq2 R package version 1.36.0 (39) for overall comparisons, e.g., treatments were compared.

## SUPPLEMENTAL MATERIAL

Supplemental_Figures spectrum.01978-23-s0001.pdf Figures S1-S10.

Supplemental_Tables spectrum.01978-23-s0002.xlsx Tables S1-S7.

ASM does not own the copyrights to Supplemental Material that may be linked to, or accessed through, an article. The authors have granted ASM a non-exclusive, world-wide license to publish the Supplemental Material files. Please contact the corresponding author directly for reuse.
