# Supplementary material for: Manipulating Agaricus bisporus developmental patterns by passaging microbial communities in complex substrates
Source: Microbiol Spectr. 2023 Oct 13;11(6):e01978-23. doi: 10.1128/spectrum.01978-23 (PMC10714785; doi:10.1128/spectrum.01978-23)
Supplement: Supplemental_Figures — Figures S1-S10. [file spectrum.01978-23-s0001.pdf]

Journal: **Microbiology Spectrum**

Title: **Manipulating *Agaricus bisporus* developmental patterns by passaging microbial communities in complex substrates**

Running title: **Manipulating fungal developmental patterns**

Fabricio Rocha Vieira[1], Isako Di Tomassi[1,2], Eoin O'Connor[1,2], Carolee T. Bull[1,2], John A. Pecchia[1], Kevin L. Hockett[1,2,3]

[1] Department of Plant Pathology and Environmental Microbiology, The Pennsylvanian State University, University Park, Pennsylvania, United States.

[2] Microbiome Center, The Pennsylvania State University, University Park, Pennsylvania, United States.

[3] The Huck Institutes of the Life Sciences, The Pennsylvania State University, University Park, Pennsylvania, United States.

Correspondent authors: (F.R.V.) vieira.cogu@gmail.com, (K.L.H.) klh450@psu.edu

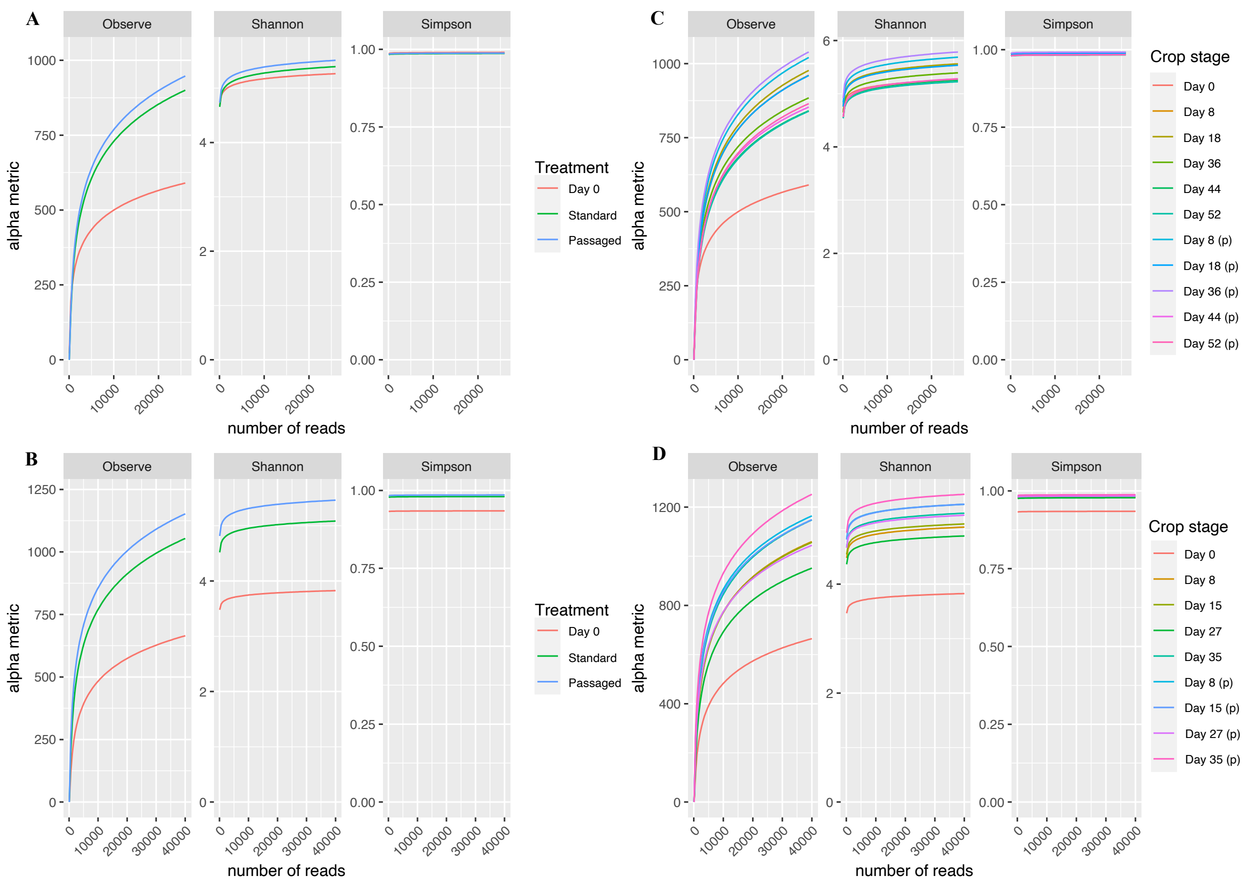

Figure S1. Rarefaction curves using alpha indexes organized by treatment/microenvironment and by crop stages. **A** - rarefaction curves for compost microenvironment using pruned and rarefied (*n* = 25,957) dataset; **B** - rarefaction curves for casing microenvironment using pruned and rarefied (*n* = 40,024) dataset; **C** - rarefaction curves by crop stages for compost microenvironment using pruned and rarefied; **D** - rarefaction curves by crop stages for casing microenvironment using pruned and rarefied dataset. In panel C and D letter "p" in parenthesis in the legend mean passaged. **Compost samples:** Day 0 – sample at the end of composting phase II in the original crop; Day 8 – sample from the middle of spawn run (day 8 after spawning) for either standard or passaged compost treatments; Day 18 – sample at the end of spawn run (16 days after spawning) for standard and passaged compost; ; Day 36 – sample from the first flush for standard and passaged compost; Day 44 – sample from the second flush for standard and passaged compost; Day 52 – sample from the third flush for standard and passaged compost. **Casing samples:** Day 0 – mixed peat moss, limestone, and case inoculum; Day 8 – sample from the middle of casehold for standard and passaged casing; Day 15 – sample from the first flush for standard and passaged casing; Day 27 – sample from the second flush for standard and passaged casing; Day 35 – sample from the third flush for standard and passaged casing. Calculations and plots were generated by the MicrobiotaProcess R package (version 1.8.1, https://github.com/YuLab-SMU/MicrobiotaProcess/).

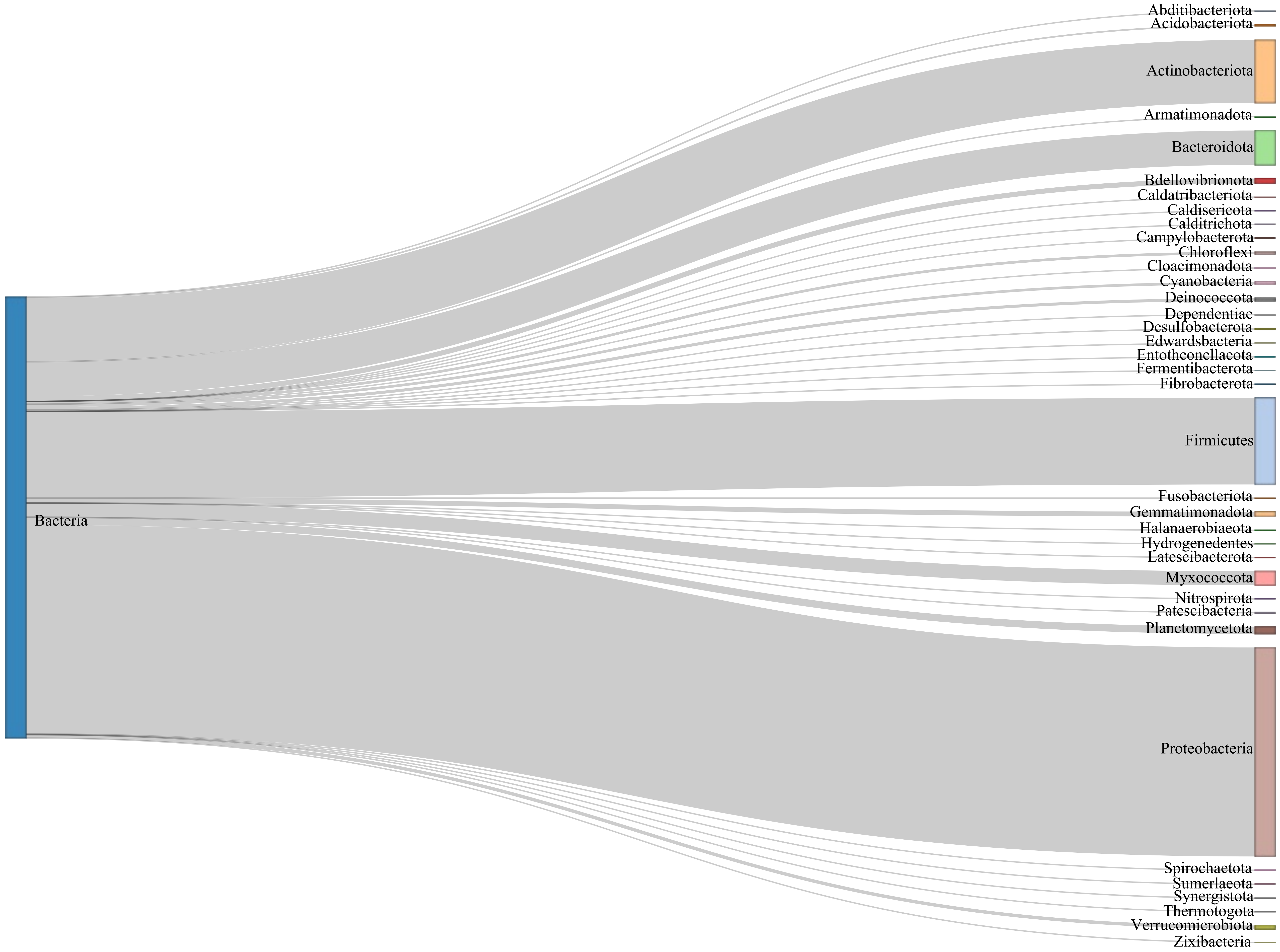

Figure S2. Absolute number of sequences sorted by phylum level for both microenvironments, compost, and casing.

```
Permutation test for adonis under reduced model
Terms added sequentially (first to last)
Permutation: free
Number of permutations: 999

adonis2(formula = unifrac.dist.psbrpcobeta ~ Treatment, data = metadf.psbrpcobeta)
          Df SumOfSqs      R2      F Pr(>F)
Treatment  2  0.14706 0.36322 8.5559  0.001 ***
Residual  30  0.25783 0.63678
Total     32  0.40489 1.00000
---
Signif. codes:  0 ‘***’ 0.001 ‘**’ 0.01 ‘*’ 0.05 ‘.’ 0.1 ‘ ’ 1
```

Figure S3. Permanova (adonis) using rarefied dataset and weighted unifrac distance for compost microenvironment sorted by treatments.

```
> pairwise.adonis(unifrac.dist.psbrpcobeta, sample_data(psbrpcobeta)$Treatment)
         pairs Df  SumsOfSqs   F.Model         R2 p.value p.adjusted sig
1 coso vs coco  1 0.11986149 10.835672 0.40377868   0.003      0.009   *
2 coso vs copa  1 0.11329415 21.349849 0.57161808   0.001      0.003   *
3 coco vs copa  1 0.02187434  2.413631 0.07936016   0.100      0.300
```

Figure S4. Pairwise adonis using rarefied dataset and weighted unifrac distance for compost microenvironment sorted by treatments. Samples (pairs): coso – compost PII; coco – standard compost and copa – passaged compost.

```
Permutation test for adonis under reduced model
Terms added sequentially (first to last)
Permutation: free
Number of permutations: 999

adonis2(formula = unifrac.dist.psbrpcobeta ~ Stage, data = metadf.psbrpcobeta)
         Df SumOfSqs      R2      F Pr(>F)
Stage    10  0.37114 0.91664 24.192  0.001 ***
Residual 22  0.03375 0.08336
Total    32  0.40489 1.00000
---
Signif. codes:  0 ‘***’ 0.001 ‘**’ 0.01 ‘*’ 0.05 ‘.’ 0.1 ‘ ’ 1
```

Figure S5. Permanova (adonis) using rarefied dataset and weighted unifrac distance for compost microenvironment sorted by crop stages.

	pairs	Df	SumsOfSqs	F.Model	R2	p.value	p.adjusted	sig
1	PII vs cossr8	1	0.056081519	37.831369	0.9043780	0.1	1	
2	PII vs cossr16	1	0.075069013	47.495103	0.9223227	0.1	1	
3	PII vs cos1	1	0.081352145	45.143768	0.9186062	0.1	1	
4	PII vs cos2	1	0.115862114	42.859835	0.9146390	0.1	1	
5	PII vs cos3	1	0.109780205	74.926838	0.9493202	0.1	1	
6	PII vs copsr8	1	0.045206987	28.081460	0.8753174	0.1	1	
7	PII vs copsr16	1	0.071385426	37.006908	0.9024555	0.1	1	
8	PII vs cop1	1	0.053047840	30.348912	0.8835480	0.1	1	
9	PII vs cop2	1	0.111024258	62.790142	0.9401109	0.1	1	
10	PII vs cop3	1	0.092695127	61.820397	0.9392286	0.1	1	
11	cossr8 vs cossr16	1	0.009822813	9.540409	0.7045880	0.1	1	
12	cossr8 vs cos1	1	0.052685201	42.110912	0.9132526	0.1	1	
13	cossr8 vs cos2	1	0.069724661	32.395162	0.8900953	0.1	1	
14	cossr8 vs cos3	1	0.087375839	95.575985	0.9598297	0.1	1	
15	cossr8 vs copsr8	1	0.030891215	29.173225	0.8794208	0.1	1	
16	cossr8 vs copsr16	1	0.041469880	30.093963	0.8826772	0.1	1	
17	cossr8 vs cop1	1	0.023626904	19.738932	0.8315004	0.1	1	
18	cossr8 vs cop2	1	0.080871718	66.439835	0.9432139	0.1	1	
19	cossr8 vs cop3	1	0.083711725	88.260325	0.9566444	0.1	1	
20	cossr16 vs cos1	1	0.034095908	25.270061	0.8633416	0.1	1	
21	cossr16 vs cos2	1	0.043554328	19.353415	0.8287188	0.1	1	
22	cossr16 vs cos3	1	0.061894721	61.139150	0.9385930	0.1	1	
23	cossr16 vs copsr8	1	0.022554921	19.493562	0.8297406	0.1	1	
24	cossr16 vs copsr16	1	0.025948388	17.578201	0.8146277	0.1	1	
25	cossr16 vs cop1	1	0.015896437	12.274055	0.7542100	0.1	1	
26	cossr16 vs cop2	1	0.055547607	42.229558	0.9134753	0.1	1	
27	cossr16 vs cop3	1	0.060964963	58.249422	0.9357424	0.1	1	
28	cos1 vs cos2	1	0.013288898	5.375817	0.5733705	0.1	1	
29	cos1 vs cos3	1	0.014099475	11.427098	0.7407160	0.1	1	
30	cos1 vs copsr8	1	0.013944661	10.115459	0.7166227	0.1	1	
31	cos1 vs copsr16	1	0.004703282	2.770428	0.4091954	0.1	1	
32	cos1 vs cop1	1	0.016547614	10.910778	0.7317377	0.1	1	
33	cos1 vs cop2	1	0.013783382	8.968432	0.6915587	0.1	1	
34	cos1 vs cop3	1	0.012359356	9.746172	0.7090099	0.1	1	
35	cos2 vs cos3	1	0.006260710	2.932314	0.4229921	0.1	1	
36	cos2 vs copsr8	1	0.028259304	12.395731	0.7560341	0.1	1	
37	cos2 vs copsr16	1	0.013261772	5.102870	0.5605782	0.1	1	
38	cos2 vs cop1	1	0.026594841	10.999415	0.7333229	0.1	1	
39	cos2 vs cop2	1	0.004487197	1.840457	0.3151220	0.2	1	
40	cos2 vs cop3	1	0.008897774	4.101613	0.5062711	0.1	1	
41	cos3 vs copsr8	1	0.030630427	29.405772	0.8802602	0.1	1	
42	cos3 vs copsr16	1	0.014678786	10.787112	0.7294942	0.1	1	
43	cos3 vs cop1	1	0.032559927	27.599539	0.8734159	0.1	1	
44	cos3 vs cop2	1	0.003585736	2.988176	0.4276046	0.1	1	
45	cos3 vs cop3	1	0.003782373	4.061734	0.5038288	0.1	1	
46	copsr8 vs copsr16	1	0.010028456	6.661402	0.6248148	0.1	1	
47	copsr8 vs cop1	1	0.004774348	3.604877	0.4740217	0.1	1	
48	copsr8 vs cop2	1	0.027280342	20.287892	0.8353089	0.1	1	
49	copsr8 vs cop3	1	0.024293703	22.579728	0.8495094	0.1	1	
50	copsr16 vs cop1	1	0.009799312	5.962329	0.5984875	0.1	1	
51	copsr16 vs cop2	1	0.014241117	8.559469	0.6815152	0.1	1	
52	copsr16 vs cop3	1	0.012705312	9.107544	0.6948322	0.1	1	
53	cop1 vs cop2	1	0.030064966	20.276605	0.8352323	0.1	1	
54	cop1 vs cop3	1	0.029399067	24.216931	0.8582411	0.1	1	
55	cop2 vs cop3	1	0.004799326	3.888500	0.4929328	0.1	1	

Figure S6. Pairwise adonis using rarefied dataset and weighted unifrac distance for compost microenvironment sorted by crop stages. Samples: PII – sample at the end of composting phase II; copsr8 – sample from the middle of spawn run for passaged compost; cossr8 – sample from the middle of spawn run for standard compost; copsr16 – sample at the of spawn run for passaged compost; cossr16 – sample at the of spawn run for standard compost; cop1 – sample from the first flush for passaged compost; cos1 – sample from the first flush for standard compost; cop2 – sample from the second flush for passaged compost; cos2 – sample from the second flush for standard compost; cop3 – sample from the third flush for passaged compost and; cos3 – sample from the third flush for standard compost.

```
> permanova <- adonis2(unifrac.dist.psbrpcabeta ~ Treatment, data = metadf.psbrpcabeta)
> permanova
Permutation test for adonis under reduced model
Terms added sequentially (first to last)
Permutation: free
Number of permutations: 999

adonis2(formula = unifrac.dist.psbrpcabeta ~ Treatment, data = metadf.psbrpcabeta)
          Df SumOfSqs      R2      F Pr(>F)
Treatment  2  0.60957 0.79453 46.403  0.001 ***
Residual  24  0.15764 0.20547
Total     26  0.76721 1.00000
---
Signif. codes:  0 ‘***’ 0.001 ‘**’ 0.01 ‘*’ 0.05 ‘.’ 0.1 ‘ ’ 1
```

Figure S7. Permanova (adonis) using rarefied dataset and weighted unifrac distance for casing microenvironment sorted by treatments.

```
> pairwise.adonis(unifrac.dist.psbrpcabeta, sample_data(psbrpcabeta)$Treatment)
         pairs Df SumsOfSqs  F.Model        R2 p.value p.adjusted sig
1 caco vs capa  1 0.1851295 29.33222 0.5714192   0.001      0.003   *
2 caco vs caso  1 0.4076447 55.04058 0.8089376   0.001      0.003   *
3 capa vs caso  1 0.3933825 63.81050 0.8307523   0.002      0.006   *
```

Figure S8. Pairwise adonis using rarefied dataset and weighted unifrac distance for compost microenvironment sorted by treatments. Samples (pairs): caso – casing mix; caco – standard casing and capa – passaged casing.

```
> permanova <- adonis2(unifrac.dist.psbrpcabeta ~ Stage, data = metadf.psbrpcabeta)
> permanova
Permutation test for adonis under reduced model
Terms added sequentially (first to last)
Permutation: free
Number of permutations: 999

adonis2(formula = unifrac.dist.psbrpcabeta ~ Stage, data = metadf.psbrpcabeta)
         Df SumOfSqs      R2      F Pr(>F)
Stage     8  0.73142 0.95335 45.983  0.001 ***
Residual 18  0.03579 0.04665
Total    26  0.76721 1.00000
---
Signif. codes:  0 ‘***’ 0.001 ‘**’ 0.01 ‘*’ 0.05 ‘.’ 0.1 ‘ ’ 1
```

Figure S9. Permanova (adonis) using rarefied dataset and weighted unifrac distance for casing microenvironment sorted by crop stages.

	pairs	Df	SumsOfSqs	F.Model	R2	p.value	p.adjusted	sig
1	cash8 vs cas1	1	0.018209272	15.558492	0.7954853	0.1	1	
2	cash8 vs cas2	1	0.042629822	35.933715	0.8998340	0.1	1	
3	cash8 vs cas3	1	0.042630904	40.978836	0.9110693	0.1	1	
4	cash8 vs caph8	1	0.040892668	31.422749	0.8870782	0.1	1	
5	cash8 vs cap1	1	0.060913029	52.895078	0.9296951	0.1	1	
6	cash8 vs cap2	1	0.084406720	56.124185	0.9334710	0.1	1	
7	cash8 vs cap3	1	0.081554966	80.720229	0.9527858	0.1	1	
8	cash8 vs ca	1	0.194752119	36.185226	0.9004609	0.1	1	
9	cas1 vs cas2	1	0.012746695	12.933508	0.7637820	0.1	1	
10	cas1 vs cas3	1	0.013834094	16.478479	0.8046730	0.1	1	
11	cas1 vs caph8	1	0.056393802	51.240018	0.9275887	0.1	1	
12	cas1 vs cap1	1	0.050034997	52.624548	0.9293593	0.1	1	
13	cas1 vs cap2	1	0.064432192	49.443893	0.9251552	0.1	1	
14	cas1 vs cap3	1	0.063458553	78.387354	0.9514489	0.1	1	
15	cas1 vs ca	1	0.268195335	51.762179	0.9282668	0.1	1	
16	cas2 vs cas3	1	0.008732846	10.207935	0.7184672	0.1	1	
17	cas2 vs caph8	1	0.077595522	69.495653	0.9455750	0.1	1	
18	cas2 vs cap1	1	0.063184623	65.356896	0.9423273	0.1	1	
19	cas2 vs cap2	1	0.061315146	46.482263	0.9207642	0.1	1	
20	cas2 vs cap3	1	0.069812017	84.567135	0.9548365	0.1	1	
21	cas2 vs ca	1	0.299418720	57.610768	0.9350763	0.1	1	
22	cas3 vs caph8	1	0.064664275	66.628397	0.9433656	0.1	1	
23	cas3 vs cap1	1	0.048321523	58.876130	0.9363829	0.1	1	
24	cas3 vs cap2	1	0.054981717	46.869635	0.9213676	0.1	1	
25	cas3 vs cap3	1	0.042254823	62.186001	0.9395643	0.1	1	
26	cas3 vs ca	1	0.291441387	57.697008	0.9351670	0.1	1	
27	caph8 vs cap1	1	0.012680815	11.722086	0.7455808	0.1	1	
28	caph8 vs cap2	1	0.027819585	19.398179	0.8290465	0.1	1	
29	caph8 vs cap3	1	0.031991022	34.013202	0.8947734	0.1	1	
30	caph8 vs ca	1	0.191560452	36.059826	0.9001493	0.1	1	
31	cap1 vs cap2	1	0.010031262	7.810413	0.6613158	0.1	1	
32	cap1 vs cap3	1	0.013424911	16.977271	0.8093174	0.1	1	
33	cap1 vs ca	1	0.249410256	48.311856	0.9235355	0.1	1	
34	cap2 vs cap3	1	0.008967457	7.844834	0.6623000	0.1	1	
35	cap2 vs ca	1	0.287911205	52.206517	0.9288339	0.1	1	
36	cap3 vs ca	1	0.280803035	55.922766	0.9332474	0.1	1	

Figure S10. Pairwise adonis using rarefied dataset and weighted unifrac distance for casing microenvironment sorted by crop stages. Samples: ca – mixed peat moss, limestone, and case inoculum; caph8 – sample from the middle of casehold for passaged casing; cash8 - sample from the middle of casehold for standard casing; cap1 – sample from the first flush for passaged casing; cas1 – sample from the first flush for standard casing; cap2 – sample from the second flush for passaged casing; cas2 – sample from the second flush for standard casing; cap3 – sample from the third flush for passaged casing and cas3 – sample from the third flush for standard casing.
